# Visual gamma oscillations predict sensory sensitivity in females as they do in males

**DOI:** 10.1038/s41598-021-91381-2

**Published:** 2021-06-08

**Authors:** Viktoriya O. Manyukhina, Ekaterina N. Rostovtseva, Andrey O. Prokofyev, Tatiana S. Obukhova, Justin F. Schneiderman, Tatiana A. Stroganova, Elena V. Orekhova

**Affiliations:** 1 Center for Neurocognitive Research (MEG Center), Moscow State University of Psychology and Education, Moscow, Russian Federation; 2 National Research University Higher School of Economics, Moscow, Russian Federation; 3 MedTech West and the Institute of Neuroscience and Physiology, Sahlgrenska Academy, The University of Gothenburg, Gothenburg, Sweden

**Keywords:** Neuroscience, Psychology

## Abstract

Gamma oscillations are driven by local cortical excitatory (E)–inhibitory (I) loops and may help to characterize neural processing involving excitatory-inhibitory interactions. In the visual cortex reliable gamma oscillations can be recorded with magnetoencephalography (MEG) in the majority of individuals, which makes visual gamma an attractive candidate for biomarkers of brain disorders associated with E/I imbalance. Little is known, however, about if/how these oscillations reflect individual differences in neural excitability and associated sensory/perceptual phenomena. The power of visual gamma response (GR) changes nonlinearly with increasing stimulation intensity: it increases with transition from static to slowly drifting high-contrast grating and then attenuates with further increase in the drift rate. In a recent MEG study we found that the *GR attenuation* predicted sensitivity to sensory stimuli in everyday life in neurotypical adult men and in men with autism spectrum disorders. Here, we replicated these results in neurotypical female participants. The *GR enhancement* with transition from static to slowly drifting grating did not correlate significantly with the sensory sensitivity measures. These findings suggest that weak velocity-related attenuation of the GR is a reliable neural concomitant of visual hypersensitivity and that the degree of GR attenuation may provide useful information about E/I balance in the visual cortex.

## Introduction

Abnormally regulated inhibitory (I) and excitatory (E) neurotransmission are implicated in the pathogenetic mechanisms of many neuropsychiatric disorders. In particular, it has been suggested that the atypically elevated E/I ratio in patients with autism spectrum disorders and/or fragile X syndrome may explain their over-responsiveness to sensory stimuli in different modalities^1–5^. Considering the proposed link between neural hyper-excitability and sensory sensitivity, the neural correlates of the latter are potentially useful noninvasive biomarkers of E/I imbalance for clinical neuroscience.

In the previous study on males, we found a link between elevated sensory sensitivity and the properties of magnetoencephalographic (MEG) gamma oscillations (~ 30–90 Hz) measured in the visual cortex^6^. Interestingly, this link was present in both autistic and neurotypical males, and therefore may reflect some basic mechanism of visual cortex function.

Gamma oscillations are generated due to the coordinated activity of excitatory and inhibitory neurons and could potentially help to characterize an altered balance between neural excitation and inhibition in brain disorders^7–11^. In humans, gamma oscillations can be reliably detected with magnetoencephalography (MEG) and are especially prominent in the visual cortex. The sensitivity of visually induced gamma oscillations to changes in neural excitation and inhibition has support from pharmacological studies^12,13^. In addition, visual gamma is characterized by high reliability^14,15^ and heritability^16^, thus fulfilling the basic requirements for neural biomarkers.

The association between parameters of visual gamma and the neural E/I balance, however, is not straightforward. Despite an initial encouraging report^17^, larger follow-up studies failed to find associations between amplitude or frequency of the visual gamma response (GR) induced by static gratings and concentration of GABA and glutamate assessed in the visual cortex using magnetic resonance spectroscopy^18,19^. Moreover, a study on patients with photosensitive epilepsy—a disorder characterized by extremely high excitability of the visual cortex—failed to detect statistically significant group differences between patients and control individuals in either amplitude or frequency of the visual GR induced by static gratings^20^.

There might be at least two reasons for these negative findings. The first one is the large inter-individual variability of induced GRs, which can be explained by morphological differences^21–23^, noise^24^, or other factors not directly related to the E/I balance. The second is the strong stimulation-dependency of gamma oscillations, which may vary in a subject-specific way. An individual may have a relatively high-power GR as compared to other individuals in the group in one experimental condition and an average or even below average GR in another condition. Therefore, gamma measured in a single condition (e.g. ‘static’) does not sufficiently characterize the general ‘propensity’ of the neural network to generate gamma oscillations. In a recent study we report a lack of significant correlation between the power of GRs induced by static and fast-moving (6°/sec) visual gratings^25^, despite a high intra-individual reliability of the GRs to both types of stimuli^26^. This strong dependency of individual GR parameters on the specific characteristics of the visual input may hinder attempts to unravel their relationships with other measures of the E/I balance.

In a series of recent studies we described the modulation of GR amplitude and frequency by changes in motion velocity of large high-contrast gratings^25–27^. In the transition from static to slow, and then to fast, visual motion resulted in *bell-shaped* changes in the GR power: it increased from the static to slow motion condition and then decreased with further increase in grating’s drift rate. By jointly modulating the grating’s contrast and velocity, we have shown that these bell-shaped changes in gamma power can be explained by changes in the strength of excitatory drive^25^.

Studies in animals have shown that growing synaptic excitation in the visual cortex in response to intensive visual stimulation is compensated by an even sharper increase in synaptic inhibition^28,29^. The nonlinear changes in the human GR with increasing excitatory drive may therefore reflect such disproportional changes in excitation vs. inhibition strength in the visual cortex. A quantitative index of individual variability in intensity-related GR attenuation—‘Gamma Suppression Slope’ (GSS)—has been found to be individually stable across measurements separated by days or weeks^26^.

These considerations suggest that *modulation* of the GR by intensity of visual stimulation may provide valuable information regarding the regulation of the E/I balance through characterizing gain control in the underlying neural network. Moreover, amplitude of the visual GR depends on the folding of the primary visual cortex and distance between cortical sources of the GR and the MEG sensors^30^. It is furthermore strongly affected by myogenic artifacts^24^. Intensity-related modulations of the GR power are less dependent on these non-physiological factors and therefore may better reflect genuine inter-individual differences in neural activity.

If GSS is sensitive to individual differences in cortical excitability, then it may also reflect their perceptual correlates, such as differences in sensory sensitivity^31–33^. Indeed, we have previously found that a less prominent attenuation of the GR (i.e. less negative GSS) correlated with sensory hypersensitivity in two independent samples of adult male participants: those with and without autism spectrum disorders^6^.

In that previous study in males we employed only moving visual gratings, and quantified individual variation in gain control in the visual cortex through the degree of velocity-related GR *attenuation* at the ‘descending branch’ of the bell-shaped curve, which describes the dependency of the GR on the grating’s drift rate at high drift velocities, i.e., when the excitatory drive to the visual cortex was high. However, the ‘ascending branch’ of the curve may also be informative. Indeed, in some patients with photosensitive epilepsy, static high-contrast gratings induce very strong GR^20,34^ and provoke seizures^34^. It is therefore conceivable that in the more excitable visual cortex the maximal GR will be attained at a less intensive excitatory drive. This individual trait might be captured by changes in the GRs with transition from static to slowly moving grating.

The present study has two aims. *First,* we sought to replicate in women the links between velocity-related attenuation of the visual GR and sensory sensitivity previously found in men^28^. Replication of this correlation in female participants would suggest that it is not gender-specific, but that relatively weak GR suppression is a ubiquitous neural concomitant of sensory hypersensitivity in general. Replication of results is furthermore important considering the general ‘replication crisis’ in cognitive neuroscience research^35,36^. *Second*, we wanted to investigate if a change in the GR at lower stimulation intensities—with transition from the static to slowly moving high-contrast grating—is also associated with sensory sensitivity. This has not been investigated in male or female participants previously.

## Methods

### Participants

Twenty seven healthy females from 18 to 39 years of age (27.63 ± 6.03 years) with normal or corrected vision were recruited for an experiment among participants of the ‘healthy-lifestyle’ Internet group (N = 15) and students (N = 12). Exclusion criteria were the presence of a psychiatric disorder, smoking, irregular menstrual cycle, and treatment with hormonal therapy. The subjects also participated in a study of the effect of menstrual cycle on brain oscillations. Therefore, MEG data were recorded twice on each individual, during follicular and luteal phases, with an interval between recordings of 5 to 147 days. For the purpose of the present study, the results were averaged over the two visits. The investigations were conducted in accordance with the Declaration of Helsinki and were approved by the Ethical Committee of the Moscow University of Psychology and Education. All participants provided verbal assent to participate in the study and were informed about their right to withdraw from the study at any time during testing. They also gave written informed consent after the experimental procedures had been fully explained.

### Adolescent/Adult Sensory Profile

To estimate subject’s sensory sensitivity in everyday life, the participants were asked to fill in the Russian version of the Adolescent/Adult Sensory Profile (A/ASP)^37^. The A/ASP allows assessing subject’s sensory processing according to the Dunn’s four quadrants model: ‘Sensory Sensitivity’, ‘Low Registration’, ‘Sensory Seeking’, and ‘Sensory Avoidance’. Apart from the ‘quadrants’, the A/ASP allows us to calculate ‘Low Neurological Thresholds’ that measure a person’s notice of, or annoyance with, sensory stimuli of different modalities. As our primary aim was to replicate our previous result obtained in males, we used the same A/ASP scales as in the initial study^6^: (1) ‘Sensory Sensitivity’ A/ASP category, that measures passive behavioral responses that characterize an individual’s sensitivity to environmental events; (2) ‘Low Neurological Threshold’ in visual modality (hereafter ‘Visual Low Threshold’), that measures a person's notice of, or annoyance with, visual stimuli. Besides, as in the previous study, we calculated (3) ‘Visual Motion Sensitivity’ based on the two A/ASP questions that measured subject's discomfort associated with intensive visual motion.

### Experimental procedure

**Figure 1:**
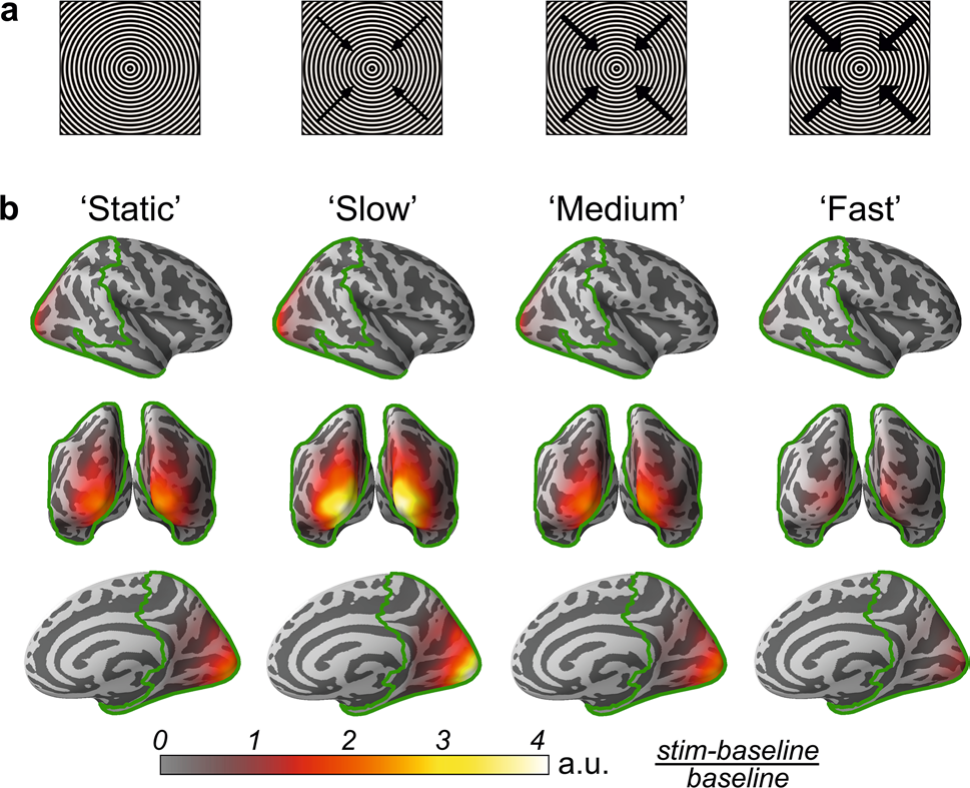
The gratings used to induce the visual gamma response (GR) (**a**) and source localization of the grand average GR power for each stimulus type (**b**). ‘Static’, ‘slow’ (1.2°/s), ‘medium’ (3.6°/s), ‘fast’ (6.0°/s) refer to the gratings’ drift rate; the arrows indicate the direction, and their thickness indicates the relative velocity of the visual motion. Color-coded cortical maps depict baseline-normalized GR power: (GR_stimulation_ – GR_baseline_)/GR_baseline_. Green borders outline the combined visual parcels used for source localization.

The experimental procedure was previously described in details^26^. In short, the participants were presented with large (18° of visual angle) high-contrast circular gratings (see Fig. 1a) that remained static (0°/s) or moved with one of three velocities: 1.2°/s (‘slow’), 3.6°/s (‘medium’), 6.0°/s (‘fast’) for 1.2–3 s. Each trial started with a fixation cross which lasted for 1.2 s and was followed by presentation of a grating of one of the four types in a random order. The participants were asked to respond to stimulation changes (motion of initially static grating or cessation of motion for the moving gratings) by pressing a button. The number of omission and commission errors (> 1000 ms and < 150 ms relative to the target event, respectively) was low (mean = 4.1%, std = 2.7%), suggesting that participants attended to the task most of the time. In total, 90 gratings of each type were presented to each individual. A short cartoon movie was presented after each 2–5 grating stimuli in order to maintain subject’s attention and decrease fatigue.

### MRI data acquisition and processing

Structural MRI data were acquired from all the participants. T1-weighted images with voxel size 1 mm^3^ were preprocessed and processed with the default algorithm (‘recon-all’) implemented in FreeSurfer software (v.6.0.0)^38^. The key processing steps therefore included motion correction, spatial normalization, skull stripping and gray/white matter segmentation.

### MEG data acquisition and preprocessing

MEG data were recorded at the Moscow Center for Neurocognitive Research (MEG-center) with an Electa VectorView Neuromag 306-channel MEG detector array (Helsinki, Finland). The MEG signal was registered with 0.03 Hz high-pass and 300 Hz low-pass inbuilt filters and sampled at 1000 Hz. The signal was then preprocessed using MaxFilter software (v.2.2) in order to reduce external noise using the temporal signal-space separation method (tSSS)^39^ and to compensate for head movements. Further preprocessing steps were performed using MNE-python software (v.0.19)^40^. The data were resampled at 500 Hz, and after that correction of biological artifacts, such as heartbeats and vertical eye movements was performed with the help of independent component analysis. On average, about two components for each MEG recording were excluded (from 1 to 4, mean: 2.16 ± 0.52). The raw data were then band-pass filtered at 30–115 Hz and epoched with a time window of − 1 to 1.2 s relative to the stimulus onset. We then excluded those epochs that contained bursts of myogenic activity or other high-amplitude artifacts, based on visual inspection of unfiltered epochs. The number of ‘good’ epochs averaged across subjects and visits was 81.64, 82.62, 81.12 and 81.95 for ‘static’, ‘slow’, ‘medium’ and ‘fast’ conditions, respectively.

### MEG source analysis

Coregistration of MEG and structural MRI data was performed using the mne_analyze tool from the MNE-C software^41^. To estimate source activations, a one-layer BEM model was created using the watershed algorithm^42^ from FreeSurfer software (v.6.0.0) with default parameters. A surface-based source space with 4096 vertices in each hemisphere was created, and then the forward model was estimated. For localization of the GR we used the Linearly Constrained Minimum Variance (LCMV) beamformer^43^. The inverse solution was limited to the cortical areas belonging to the visual cortex and related cortical areas based on the Desikan-Killiany atlas^44^: the bank of the superior temporal gyrus, cuneus, precuneus, fusiform gyrus, inferior and superior parietal gyri, lateral occipital gyrus, lingual gyrus, pericalcarine gyrus. Since we expected that the spatial maxima of GRs may differ for stimuli moving at different velocities, the LCMV spatial filters were created separately for each of the stimulation conditions. Noise covariance matrices were estimated for the baseline intervals (− 0.9 to 0 s) of all epochs, while the data covariance matrices were estimated for the stimulation intervals (0.3–1.2 s), individually for each of four types of stimuli/epochs (‘static’, ‘slow’, ‘medium’, ‘fast’). The LCMV filters for each stimulus/epoch group were applied to each epoch individually, with a regularization coefficient of 0.05.

Time–frequency analysis was performed in source space, using the multi-taper method (bandwidth = 10 Hz, frequency resolution ~  = 1.11 Hz, time step = 2 ms), separately for conditions and time intervals (baseline and stimulation).

The normalized response power was estimated in each vertex and frequency bin as (stimulation—baseline)/ baseline, where ‘baseline’ and ‘stimulation’ correspond to the signal power in (− 0.9 to 0 s) and (0.3–1.2 s) time intervals, respectively. We then identified 26 vertices with maximal normalized response power in 40–80 Hz range. This was done separately for each subject, stimulus/epoch type (‘static’, ‘slow’, ‘medium’, ‘fast’) and visit (1st, 2nd).

The mean normalized gamma-range (30–115 Hz) power spectra over 26 vertices were then smoothed with a moving average (step = 3). Finally, for each of the stimuli/epoch types, we estimated the weighted GR power and frequency. The weighted GR power was calculated as the average over those power values, which exceed 2/3 of the GR peak in the 35–90 Hz range. The weighted GR frequency was estimated as the center of gravity over the frequencies corresponding to power values selected for the weighted GR power calculation. We then estimated the reliability of the GRs using a strategy applied in our previous studies^27^. In brief, the probability of a power difference in the baseline and stimulation intervals was estimated at the single trial level. Only subjects who had reliable GRs (Wilcoxon rank test: p < 0.0001 at the ‘maximal power’ frequency bin) in the stimulus condition characterized by maximal GR (usually the ‘slow’ condition) during both visits were included in the analysis. All 27 participants fulfilled this criterion.

To quantify stimulation-related changes in the GR power, we used two indices. One of them quantified the attenuation of the GR with increasing motion velocity (‘descending branch’ of the bell-shaped curve that characterizes the dependency of the GR on motion velocity of the grating). Another one estimated changes in the GR between ‘static’ and ‘slow’ stimulation conditions (i.e. at the ‘ascending branch’).

The attenuation of the GR to moving high-contrast circular gratings caused by increasing their motion velocity (from 1.2 to 6.0°/s) is a robust phenomenon that has been previously described in multiple studies^6,25–27,45^. To quantify this attenuation, we have previously introduced the Gamma Suppression Slope (GSS) index^6^. In order to estimate the GSS, the GR power was normalized to the subject’s GR power in the ‘slow’ condition and the GSS was calculated as the coefficient of linear regression of the normalized GR power (y) to velocity (x), while setting the intercept to zero (implemented with the Matlab function ‘fitlm’):

$${\text{GSS }} = {\text{fitlm}}\left( {{\text{x}},{\text{ y}}, \, {\text{'Intercept'}},{\text{ false}}} \right)$$

This regression coefficient is proportionally more negative in case of stronger attenuation of the GR with increasing motion velocity.

Enhancement of the GR with a moderate increase of high-contrast grating’s motion velocity (up to ~  <  = 1.33°/s) is a repeatedly reported finding^25–27,46–48^. Here we introduced the Gamma Enhancement Index (GEI) as the normalized differences in the GR power in the ‘static’ and ‘slow’ conditions:

$${\text{GEI }} = \, \left( {{\text{GR}}_{{{\text{slow}}}} - {\text{GR}}_{{{\text{static}}}} } \right)/{\text{GR}}_{{{\text{slow}}}}$$

### Statistical analysis

Statistical analysis was performed using Numpy and Scipy libraries in Python 3.7. To be able to compare the results of this study in women with the results of the previous study in men^6^, similarly to the previous study, we used Spearman’s rank correlation coefficient to assess the link between gamma-based measures (GSS, GEI) and A/ASP scales.

## Results

### Adolescent/Adult Sensory Profile (A/ASP)

For comparison with the previous study we were interested specifically in the ‘Sensory Sensitivity’ quadrant of the A/ASP that measures passive behavioral responses to sensory stimulation, such as noticing behaviors, distractibility, and discomfort with sensory stimuli. However, to ensure general validity of the A/ASP data obtained in our female sample using Russian translation of the A/ASP, we compared all the A/ASP main scores (‘quadrant’) with those provided in the original manual^37^ (Table 1). The means of these scores, including the ‘Sensory Sensitivity’ fall into the normative range. The individual ‘Sensory Sensitivity’ scores ranged from 25 (‘less than most people’, i.e. < 16% of people according to^37^) to 50 (‘much more than in most people’, i.e. > 98% of people according to^37^). In the present study, however, the individual scores have to be treated with caution, because the questionnaire has not been standardized for the Russian sample. Nevertheless, these scores suggest considerable inter-individual variability in Sensory Sensitivity in our female sample.

**Table 1:** Adolescent/Adult Sensory Profile (A/ASP) quadrant scores: comparison with the normative data.

A/ASP quadrant	Current sample (N = 27) Range	Current sample (N = 27) Mean ± SD	‘Similar to most people’ (68%) range^32^
Low registration	24–45	31.04 ± 4.76	24–35
Sensory seeking	30–64	47.37 ± 8.04	43–56
Sensory sensitivity	25–50	37.74 ± 6.48	26–41
Sensory avoiding	28–54	40.22 ± 7.68	27–41

The ‘Visual Low Threshold’ in our participants ranged from 7 to 25 (mean = 14.7, SD = 4.4). The ‘Visual Motion [Low] Threshold’ ranged from 2 to 9 (mean = 4.9, SD = 1.8). Supplementary Table S1 provides ‘Low Thresholds’ A/ASP scores for all modalities for the female participants in the present, as well as for the male participants in our previous, study^6^.

### GR localization in the source space

The group average source localizations of the GRs induced by the static and moving gratings are presented in Fig. 1b. In all participants the GRs in the ‘maximal gamma’ condition were reliable (p < 0.0001) during both visits. All participants were therefore included in the analysis.

In the majority of participants, the vertex source with the maximal GR power (‘maximal vertex’) was localized to the pericalcarine sulcus or lateral occipital sulcus (i.e., in the primary visual cortex), irrespective of the stimulus type and hemisphere. According to the Desikan-Killiany atlas^44^, the group average MNI coordinates belonged to an area near the border between the pericalcarine sulcus and lateral occipital sulcus in both hemispheres (see Supplementary Table S2 for group average MNI coordinates of the ‘maximal vertex’, separately for condition and brain hemisphere).

### Velocity-related changes in GR power

**Figure 2:**
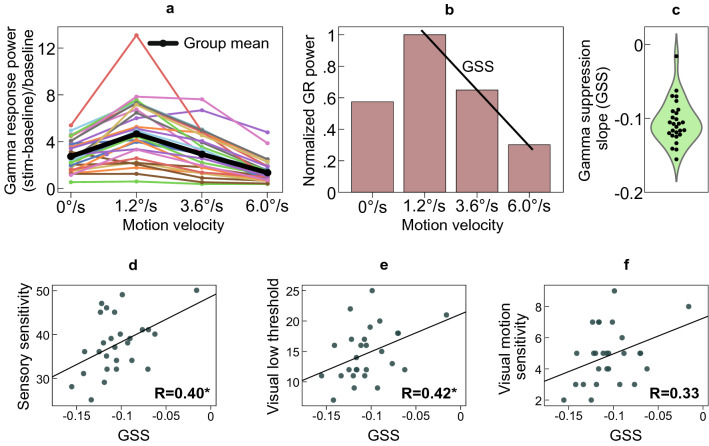
Attenuation of the gamma response (GR) power with increasing motion velocity of the grating (i.e., the Gamma Suppression Slope, GSS) and its correlations with Adolescent/Adult Sensory Profile (A/ASP) scales. (**a**) Lines representing the changes in the GR power at each velocity and for each participant, as well as the group mean (thick black line). (**b**) Group average GR power normalized to the ‘maximal GR’ (usually GR in the ‘slow’ condition). The line illustrates the GSS that quantifies the magnitude of velocity-related GR attenuation. (**c**) Violin plot of the GSS values. (**d**–**f**) Correlations between the GSS index and three A/ASP sensitivity scales: (**d**) ‘Sensory Sensitivity’, (**e**) ‘Visual Low Threshold’, (**f**) ‘Visual Motion Sensitivity’. R’s are Spearman rank correlation coefficients, *p < 0.05.

Bell-shaped changes in GR power caused by increasing the velocity of visual motion have been reported in our previous studies^25–27^. Here, we present these data to illustrate between-subjects variability in GR power in the current female sample (Fig. 2a). In 26 of 27 subjects the GR power increased from the ‘static’ to ‘slow’ visual motion conditions. In the remaining subject the GR power was maximal in the static condition (for this subject, the relative GR power in static/slow conditions was 3.1/2.1). In one participant the maximal GR was attained at the ‘medium’ velocity. In the rest of the subjects (25 of 27) the maximal GR was observed at the ‘slow’ velocity.

### A/ASP and GR attenuation with transition from slowly to rapidly moving gratings

The GSS (see Fig. 2b for the group average GSS and Fig. 2c for the GSS distribution) directly correlated with general Sensory Sensitivity (Spearman R_(27)_ = 0.40, p = 0.038; Fig. 2d) and with Visual Low Threshold (Spearman R_(27)_ = 0.42, p = 0.028; Fig. 2e). Neither Visual Low Threshold nor Sensory Sensitivity scales correlated with GR power or frequency measured in any particular condition (all Spearman R_(27)_ p’s > 0.07). There was also a tendency for correlation between the GSS and Visual Motion Sensitivity (Spearman R_(27)_ = 0.33, p = 0.09; Fig. 2f). These findings showed that women who reported higher sensory sensitivity to, and avoidance of, intensive sensory stimuli also had a less negative GSS index, i.e., they demonstrated a less prominent attenuation of the visual GR when the velocity of visual motion increased.

The correlations of GSS with all other A/ASP quadrants and ‘Low Threshold’ scales are given in Supplementary Table S3 together with the corresponding results from the previous study in males^6^. Of note, the Visual Low Threshold was the only modality specific scale that was reliably associated with GSS in both studies (p’s < 0.05).

To check for a possible contribution of attention and task engagement, we calculated correlations between GSS vs. subjects’ reaction time, percent of omission errors, and percent of commission errors. No significant correlations were found (all p’s > 0.30).

In our female participants, who were all between 18 and 39 years of age, the GR power significantly increased with age (Spearman correlation coefficient for ‘static’: R_(27)_ = 0.52, p = 0.005; ‘slow’: R_(27)_ = 0.49, p = 0.01; ‘medium’: R_(27)_ = 0.48, p = 0.01; ‘fast’: R_(27)_ = 0.47, p = 0.01), but the GSS did not change with age (R_(27)_ = 0.00, p = 0.98).

### A/ASP and GR enhancement with transition from static to moving gratings

**Figure 3:**
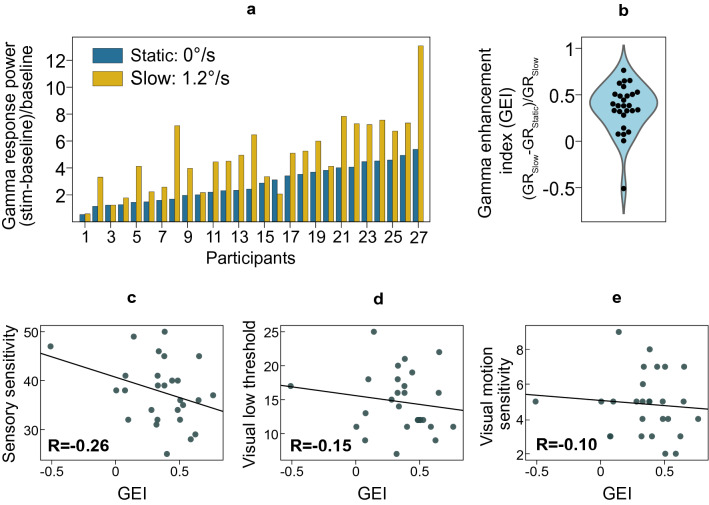
Velocity-related enhancement of the gamma response (GR) power with transition from static to slowly moving gratings (i.e., Gamma Enhancement Index, GEI) and its correlations with Adolescent/Adult Sensory Profile (A/ASP) scales. (**a**) Individual GR values in the ‘static’ and ‘slow’ motion conditions. (**b**) Violin plot of the GEI values. (**c**–**e**) Correlations between the GEI and three A/ASP sensitivity scales: (**c**) ‘Sensory Sensitivity’, (**d**) ‘Visual Low Threshold’, (**e**) ‘Visual Motion Sensitivity’. R’s are Spearman rank correlation coefficients, *p < 0.05.

While in the majority of the subjects the GR power increased from the ‘static’ to ‘slow’ stimulation condition, there was considerable individual variability, with some participants showing nearly no change or even a decrease in the GR power (Fig. 3a). We calculated correlations between GEI (normalized GR difference between ‘slow’ and ‘static’ conditions; see Fig. 3b for the GEI distribution) and the A/ASP sensitivity scores. The correlations were all not significant (GEI vs. Sensory Sensitivity: Spearman R_(27)_ = − 0.26, p = 0.20; GEI vs. Visual Low Threshold: Spearman R_(27)_ =  − 0.15, p = 0.47; GEI vs. Visual Motion Sensitivity: Spearman R_(27)_ =  − 0.10, p = 0.63; Fig. 3c–e).

To check for a possible contribution of attention and task engagement, we calculated correlations between GEI vs. subjects’ reaction time, percent of omission errors, and percent of commission errors. No significant correlations were found (all p’s > 0.26). GEI did not change with age (Spearman R_(27)_ = 0.11, p = 0.56).

## Discussion

In this study, we tested for links between neural gain control, measured with visual gamma oscillations, and subjects’ sensory sensitivity in everyday life. In a sample of neurotypical women, we replicated the correlation between the intensity-related modulations of visual gamma oscillations and sensory sensitivity, which we have previously found in men^6^. Specifically, those subjects who were more sensitive to sensory stimuli in general and especially to visual stimuli according to their subjective reports also displayed lower attenuation of the GR when the drift rate of high-contrast gratings increased beyond the ‘optimal’ (slow) velocity, i.e., the visual motion velocity that produced the maximal gamma response in the majority of participants.

Beyond replication of the previous findings on males, we also investigated the association of sensory sensitivity with changes in GR from static to slowly-moving visual gratings. In accordance with our previous^25–27^ and others’^46–48^ findings, the majority of the subjects (26 of 27) demonstrate an increase in GR power when stimulated with slowly moving gratings, as compared to static gratings. However, none of the correlations between change in the GR power at the ‘ascending branch’ of the curve and A/ASP scales of interest reached significance (all p’s >  = 0.2).

Below, we briefly discuss possible mechanisms behind these findings and argue that *intensity-related modulations* of the MEG gamma response might provide informative noninvasive indicators of excitatory-inhibitory interactions in the brain.

It has been previously shown that increasing the motion velocity of high-contrast visual grating leads to a linear increase in the frequency of MEG gamma oscillations and nonlinear—bell-shaped—changes in GR power^25–27^. The GR power increases with a moderate increase in velocity/drift rate of a grating (1.2°/cycle spatial frequency), but then attenuates at yet higher velocities. By modulating a grating’s contrast together with its velocity, we have recently shown that the nonlinear velocity-related changes in GR power can be better explained by *growing excitatory drive* to the visual cortex, rather than by tuning of the neurons to a particular velocity^25^. A similar nonlinear behavior in gamma oscillations was observed in nonhuman primates when the excitatory drive was modulated by the velocity of moving gratings^49^ or the visual contrast of a static grating^50,51^.

While an increase of the GR power with increasing excitatory drive is well explained by recruitment of a progressively greater number of excitatory and inhibitory neurons in synchronous oscillatory activity^52^, the mechanism leading to the GR attenuation at high input intensities is less obvious. Modeling studies may provide a formal description of the nonlinear behavior of gamma oscillations^53,54^, but their explanatory power is often limited by the necessity to apply constrains and assumptions that are not physiologically well-justified^55^. In this respect, results provided by animal studies are of great value.

Recent voltage-clamp measurements in awake mice by Adesnik^28^ demonstrated that an increase of the excitatory drive to the primary visual cortex via increasing visual contrast leads to a sub-linear growth of neural excitation, super-linear growth of neural inhibition, and thus a reduction in the E/I ratio. It is likely that similar changes in excitation and inhibition take place in the human primary visual cortex when the excitatory drive is modulated by the motion velocity of high-contrast gratings. Indeed, an increase in gratings’ velocity leads not only to attenuation of the GR power, but also to an increase in the GR peak frequency, which is thought to be regulated by tonic excitability of inhibitory neurons^56^. The bell-shaped changes in MEG gamma response power (Fig. 2a) may therefore result from a disproportionally higher increase of inhibition than excitation and thus changes in their ratio. The moderate input to the primary visual cortex associated with slowly moving gratings may excite the excitatory and inhibitory neurons at a level which is optimal for gamma synchronization. Further increasing the excitatory drive beyond a ‘transition point’ then leads to the attenuation of the GR through the mechanisms modeled by Borgers et al.^53^ or some other yet unknown ones. Whatever the exact mechanism, the role of growing inhibition in the GR attenuation at high levels of excitatory drive is highly plausible.

Visual hypersensitivity is thought to be associated with elevated excitability of the visual cortex^31–33^. The effect of this common underlying neural factor may explain the association between velocity-related attenuation of the GR and A/ASP visual sensitivity that we observe (Fig. 2e). The fact that, among different sensory modalities, the correlation with GSS was most reliably reproduced in the visual modality (Supplementary Table S3) is an expected result, since GSS primarily reflects neural processes in the visual cortex. The present finding thus strengthens our previous hypothesis that ‘the slope of the stimulus–response function of visual gamma may provide a tractable and accessible measure of the capacity to regulate the E/I balance in visual circuitry according to intensity of the visual input’^6^.

Since gamma synchronization enhances information transfer in cortical circuits^57–62^, it is conceivable that attenuation of the GR power with increasing motion velocity of large high-contrast gratings reduces the perceived strength of this powerful stimulation. Apart from perceptual consequences, the attenuation of gamma oscillations may reduce the potentially harmful effect of strong visual stimuli in the brain. Indeed, previous studies have suggested that gamma (30–80 Hz) oscillations in the visual cortex may play a role in seizure generation in patients with photo-sensitive epilepsy, and the same visual stimuli that induce strong gamma oscillations may provoke seizures^34^. The lack of velocity-related suppression of the GR in an epilepsy patient described in our recent study^27^ is in line with the proposed protective role of GR attenuation.

It has been previously shown that elevated neural excitability is associated with reduced neural gain control, as measured with steady-state visual evoked potentials^63,64^. In the case of gamma oscillations, the gain control deficit may result in a propensity of the cortex to generate gamma in a relatively broader range of stimulation intensities, both high and low. Therefore, higher neural excitability and greater sensory sensitivity might be associated not only with lower GR attenuation at high stimulation intensities (i.e. less negative GSS), but also with a propensity to attain a near maximal GR at already relatively lower intensities (i.e. lower GEI). We did not find support for this hypothesis. A limitation of the present study is, however, the small number of measurement points at the ‘ascending branch’ of the velocity curve (only 0 and 1.2°/s, see Fig. 2a). This may have resulted in a sub-optimal estimation of the motion-related GR enhancement and thus unreliable correlation results. In the future, this parameter could be better estimated by studying the ascending branch of the GR curve in more detail. One approach to achieve this would be to employ several motion velocities between our ‘static’ and ‘slow’ (1.2°/s) ones (e.g. 0.33, 0.6°/s, etc.; see in^48^). Another possibility is to decrease the contrast of the gratings^25^ in order to preclude GR ‘saturation’ observed in some participants already in response to the static grating.

In conclusion, we found a link between sensory sensitivity and gamma response attenuation at strong stimulation intensity in female participants, thus replicating the previous findings in males. Our results suggest that changes in gamma response power at strong stimulation intensities might be more informative regarding neural excitability of the visual cortex than those at low stimulation intensities; additional research is required to support this preliminary finding. The present study thus extends our previous results linking neural gain control measured through visual gamma oscillations to individual differences in sensory sensitivity. It suggests that the nonlinear behavior of gamma oscillations under varying sensory load may provide information about E/I balance regulation in the healthy brain and possibly under pathological conditions that are characterized by an E/I imbalance in cortical circuits.

## Supplementary Information

Supplementary Information.
