# Supplementary material for: Visual gamma oscillations predict sensory sensitivity in females as they do in males
Source: Sci Rep. 2021 Jun 8;11:12013. doi: 10.1038/s41598-021-91381-2 (PMC8187436; doi:10.1038/s41598-021-91381-2)
Supplement: Supplementary file 1 — Supplementary Information. [file 41598_2021_91381_MOESM1_ESM.doc]

**Visual gamma oscillations predict sensory sensitivity in females as they do in males**

**Supplementary Information**

Viktoriya O. Manyukhina^1 ,2^, Ekaterina N. Rostovtseva^1^ , Andrey O. Prokofyev^1^ , Tatiana S. Obukhova^1^, Justin F. Schneiderman^3^, Tatiana A. Stroganova^1^ , Elena V. Orekhova^1 ,3^*

^1^ Center for Neurocognitive Research (MEG Center), Moscow State University of Psychology and Education, Moscow, Russian Federation

^2^ National Research University Higher School of Economics, Moscow, Russian Federation

^3^ MedTech West and the Institute of Neuroscience and Physiology, Sahlgrenska Academy, The University of Gothenburg, Gothenburg, Sweden

| A/ASP item | Females (27)  mean (sd) | Males (19)  mean (sd) |
| --- | --- | --- |
| *A/ASP quadrants* | | |
| Low Registration | 31.0 (4.8) | 26.7 (7.1) |
| Sensation Seeking | 47.4 (8.0) | 48.1 (6.2) |
| Sensory Sensitivity | 37.7 (6.5) | 31.2 (7.7) |
| Sensation Avoiding | 40.2 (7.7) | 31.4 (7.2) |
| *Low Neurological Thresholds* | | |
| Taste | 8.0 (2.4) | 6.2 (2.0) |
| Movement | 8.1 (1.8) | 6.9 (1.7) |
| ***Visual*** | 14.7 (4.4) | 12.9 (3.8) |
| Touch | 18.0 (3.9) | 13.6 (4.2) |
| Activity | 12.0 (2.4) | 9.9 (2.4) |
| Auditory | 17.1 (4.6) | 13.0 (3.4) |

**Supplementary table S1.** Means and standard deviations of the Adolescent/Adult Sensory Profile (A/ASP) measures in neurotypical females in the present study and in neurotypical males in the previous study ^1^.

| **Experimental**  **condition** | **Left hemisphere** | | | **Right hemisphere** | | |
| --- | --- | --- | --- | --- | --- | --- |
|  | X | Y | Z | X | Y | Z |
| Static | -9.46 | -98.92 | -1.15 | 10.93 | -97.18 | 0.77 |
| Slow | -7.94 | -97.50 | -0.70 | 10.08 | -96.72 | 0.71 |
| Medium | -7.87 | -94.67 | 0.09 | 10.59 | -92.90 | 2.81 |
| Fast | -9.96 | -95.83 | 3.36 | 8.67 | -92.13 | 5.28 |

**Supplementary table S2.** Group average MNI coordinates of the vertices with maximal gamma response (GR).

| **A/ASP items** | **Present study in females**,  Spearman R | **Previous study in males**,  Spearman R |
| --- | --- | --- |
| *A/ASP quadrants* | | |
| Low Registration | 0.17 | 0.17 |
| Sensory Seeking | -0.10 | -0.02 |
| Sensory Sensitivity | **0.40*** | **0.5**** |
| Sensation Avoiding | 0.36^#^ | 0.23 |
| *A/ASP Low Thresholds* | | |
| Taste | -0.04 | **0.41*** |
| Movement | 0.03 | **0.33*** |
| **Visual** | **0.42*** | **0.47**** |
| Touch | 0.34^#^ | 0.27^#^ |
| Activity | 0.29 | 0.29^#^ |
| Auditory | **0.45*** | 0.26 |

^#^p<0.1, *p<0.5, **p<0.01

**Supplementary table S3.** Spearman correlations between the Adolescent/Adult Sensory Profile (A/ASP) scales and gamma suppression slope (GSS) in neurotypical females in the present study and in neurotypical males in the previous study ^1^.

**Reference**

^1^ Orekhova, E. V., Stroganova, T. A., Schneiderman, J. F., Lundstrom, S., Riaz, B., Sarovic, D., Sysoeva, O. V., Brant, G., Gillberg, C. & Hadjikhani, N. **Neural gain control measured through cortical gamma oscillations is associated with sensory sensitivity**. *Hum Brain Mapp* 2019, **40**(5):1583-1593.
